# Toward Data-Driven Radiation Oncology Using Standardized Terminology as a Starting Point: Cross-sectional Study

**DOI:** 10.2196/27550

**Published:** 2022-01-19

**Authors:** Nikola Cihoric, Eugenia Vlaskou Badra, Anna Stenger-Weisser, Daniel M Aebersold, Matea Pavic

**Affiliations:** 1 Inselspital Bern University Hospital University of Bern Bern Switzerland; 2 Department of Radiation Oncology University Hospital Zurich University of Zurich Zurich Switzerland; 3 Luzerner Kantonsspital Luzern Switzerland

**Keywords:** terminology, semantic interoperability, radiation oncology, informatics, medical informatics, oncology, lexical analysis, eHealth

## Abstract

**Background:**

The inability to seamlessly exchange information across radiation therapy ecosystems is a limiting factor in the pursuit of data-driven clinical practice. The implementation of semantic interoperability is a prerequisite for achieving the full capacity of the latest developments in personalized and precision medicine, such as mathematical modeling, advanced algorithmic information processing, and artificial intelligence approaches.

**Objective:**

This study aims to evaluate the state of terminology resources (TRs) dedicated to radiation oncology as a prerequisite for an oncology semantic ecosystem. The goal of this cross-sectional analysis is to quantify the state of the art in radiation therapy specific terminology.

**Methods:**

The Unified Medical Language System (UMLS) was searched for the following terms: *radio oncology*, *radiation oncology*, *radiation therapy*, and *radiotherapy*. We extracted 6509 unique concepts for further analysis. We conducted a quantitative analysis of available source vocabularies (SVs) and analyzed all UMLS SVs according to the route source, number, author, location of authors, license type, the lexical density of TR, and semantic types. Descriptive data are presented as numbers and percentages.

**Results:**

The concepts were distributed across 35 SVs. The median number of unique concepts per SV was 5 (range 1-5479), with 14% (5/35) of SVs containing 94.59% (6157/6509) of the concepts. The SVs were created by 29 authors, predominantly legal entities registered in the United States (25/35, 71%), followed by international organizations (6/35, 17%), legal entities registered in Australia (2/35, 6%), and the Netherlands and the United Kingdom with 3% (1/35) of authors each. Of the total 35 SVs, 16 (46%) did not have any restrictions on use, whereas for 19 (54%) of SVs, some level of restriction was required. Overall, 57% (20/35) of SVs were updated within the last 5 years. All concepts found within radiation therapy SVs were labeled with one of the 29 semantic types represented within UMLS. After removing the *stop words*, the total number of words for all SVs together was 56,219, with a median of 25 unique words per SV (range 3-50,682). The total number of unique words in all SVs was 1048, with a median of 19 unique words per vocabulary (range 3-406). The lexical density for all concepts within all SVs was 0 (0.02 rounded to 2 decimals). Median lexical density per unique SV was 0.7 (range 0.0-1.0). There were no dedicated radiation therapy SVs.

**Conclusions:**

We did not identify any dedicated TRs for radiation oncology. Current terminologies are not sufficient to cover the need of modern radiation oncology practice and research. To achieve a sufficient level of interoperability, of the creation of a new, standardized, universally accepted TR dedicated to modern radiation therapy is required.

## Introduction

### Background

It is a globally accepted concept that data-driven medicine leads to better, safer, and more affordable care. In addition, it is perceived that it is not achievable without the free exchange of data among all members of a health care enterprise [1-3]. From a technical perspective, we have witnessed significant advances in the last decade. More than 160 exabytes of data are seamlessly exchanged among different systems via the internet every day [4]. At its core, internet communication relies on standardized data formats, such as Internet Message Format, XML, and JavaScript Object Notation. Security is ensured through robust encryption algorithms and authentication services, such as the OAuth protocol. Various industries have used the advantages of digitalization to simplify, accelerate, and standardize their work processes. However, data interoperability in health care has yet to reach its full potential [3].

The surge of health care digitalization in the United States during the last decade, supported by 36 billion dollars of government stimulation packages, failed to deliver on the promise of health care advancements. Mainstream media describe the current state of eHealth care records in the United States as an “unholy mess” [5,6]. Conversely, reports on software failures, safety, and security issues in scientific journals are scarce because of entrenched secrecy policies and so-called *gag* clauses that prevent physicians and researchers from publishing [7]. Furthermore, this flawed digitalization has resulted in a dramatic rise in burnout symptoms among health care workers. At least one serious symptom of burnout is recorded in 70% of physicians, a situation that is partly attributable to problems with software usability, user-unfriendly interfaces, one-size-fits-all software approaches, and foremost—to the lack of interoperability [1,8].

Contemporary health care information systems require multiple low-level manual operations, such as copy-pasting information from one interface to another, which often results in erroneous and repetitive work. Furthermore, the lack of interoperability, along with other design issues, is recognized as one of the main reasons for preventable medical errors, preventing the efficient conduct of clinical research and medical education [9-13].

The Healthcare Information and Management Systems Society defines interoperability as the ability of different information systems, devices, and applications to access, exchange, integrate, and cooperatively use data in a coordinated manner within and across organizational, regional, and national boundaries. Furthermore, interoperability is divided into four distinct levels: foundational, structural, semantic, and organizational. The American Standard Code for Information Interchange, Unicode (an information technology standard for the consistent encoding, representation, and handling of text), World Wide Web Consortium, and Health Level Seven govern foundational and structural interoperability levels. Transfer protocols and file formats used in communication are well established and standardized [14].

Organizational interoperability concerns policies, laws, regulations, and ethical considerations that span form individual actors, across health care facilities and service providers, all the way to state and international levels. The interoperability levels mentioned above are difficult to influence within a daily clinical or research routine. Foundational and technical standards necessary for clinical data exchange are well covered by the Fast Healthcare Interoperability Resources [15], a set of rules describing data formats and elements for exchanging electronic health records. Organizational interoperability refers to the willingness and ability of organizations to transfer data, which in health care are heavily regulated by the authorities that are hard to influence (by ordinary clinicians or researchers).

The semantic interoperability (SI) level is important for patients and clinicians. SI, as defined by the Healthcare Information and Management Systems Society, is a property of systems that share data with unambiguous meanings. More precisely, SI is defined as the underlying models and codification of the data, including the use of data elements with standardized definitions from publicly available value sets and coding vocabularies, providing a shared understanding and meaning to the user [16,17]. Clinical dictionaries, terminologies, or coding systems are structured lists of terms and phrases paired with their definitions or, eventually, codes. Their purpose is to describe the care and treatment of patients unambiguously. Recently, new types of lexical resources, such as ontologies and graph analytic tools, have emerged. They play a significant role in knowledge organization and management, for example, in genome-based research or enterprise business development [18,19].

### Objective

The need for standardized communication in radiation oncology has been well recognized and described by several authors and groups [20-22]. However, little is known about the availability of dedicated radiation oncology lexical resources. This study aims to evaluate the current state of radiation oncology-specific terminology as a prerequisite for data-driven radiation oncology.

## Methods

### Goals

The primary goal of the project was to quantitatively evaluate existing radiation therapy-specific source vocabularies (SVs) available in the Unified Medical Language System (UMLS). Secondary goals were the lexical analysis of SVs and qualitative analysis, which was done to verify whether the existing terminology resources (TRs) were sufficient to cover radiation therapy needs.

### Definition of TRs

There is no clear distinction between existing terminology and lexical resources that can be acquired via literature or general internet research. For this project, we defined TR as any comprehensive resource found within UMLS SVs, such as a vocabulary, taxonomy, thesaurus, coding system, ontology, or any other type.

### Material for Research

UMLS [23] was used as the basis for this study. To the best of our knowledge, UMLS is the most comprehensive repository of biomedical terminologies developed and maintained by the United States National Library of Medicine [23]. It consists of 216 vocabulary items (last reviewed: May 4, 2020) in English (151/216, 70% of content) and other languages that contribute to a total of 15,479,756 concept names and synonyms. After individual registration and acceptance of an individual licensing agreement (by NC), UMLS-registered users can search content through an HTML, a web-based graphical user, or an application programming interface. Data records are accessible for download in JavaScript Object Notation format.

### Methodology

We aimed to cover the terms specific to radiation therapy. To maximize sensitivity and specificity we searched UMLS on June 15, 2020, for the following terms: *radiation therapy*, 6030 concepts; *radiotherapy*, 479 concepts; *radiation oncology*, 58 concepts; and *radio oncology*, 0 concepts. In total, 6567 concepts were retrieved and exported, together with metadata in the JavaScript Object Notation format. We designed a denormalized database to facilitate further analysis. For this work, we downloaded the following data points from the UMLS server:

- Unique concept identifier—a code value that uniquely identifies a single concept;
- Route Source—an entity that has authored the TR;
- Name—a string chosen to represent the concept as a whole;
- Definition of a concept:
  Atoms—the smallest unit of naming in a source (a specific string with specific code values and identifiers from a specific source);
- Semantic type—a category of a concept assigned by the UMLS.

We analyzed all SVs according to the route source, number, author, location of the author, license type, lexical density of TR, and semantic types.

The data on TR authors were searched within the UMLS website or by general internet search (via Google) if the data were not available. We recorded the country where the legal residence of the authors was registered in the official state company register. *International organization* was defined as an institution drawing membership from at least 3 states and having activities in several states.

Licensing was categorized into two main groups: free TR, where no use restriction applied, and restricted TR, where any use was limited under conditions specified in the license agreement. Further evaluation of licensing types and terms of use was beyond the scope of this study.

*Word* is defined as a combination of characters representing a spoken sound that can be uttered in isolation with objective or practical meaning. The lexical density of a TR serves as a measure of the structure and complexity of communication. It is defined as the ratio of the total number of words that describe all concepts within a TR and the number of unique words used to describe the concepts. The word propagation index was used as a measure of the importance of a word and was expressed as the number of SVs that contained this word.

Data processing and lexical analysis were performed using Python (version 3.7; Python Software Foundation) and Python library Natural Language Toolkit (version 3.5).

The systematic review of other biomedical terminology services and repositories, such as Open Biological and Biomedical Ontology Foundry [24] or BioPortal [25], was beyond the scope of this work.

## Results

### Overview

A total of 6567 concepts were retrieved from UMLS. After the removal of duplicate entries, 6509 unique concepts were selected for further analysis.

The concepts were distributed across 35 SVs. The median number of unique concepts was 5 per SV (range 1-5479 concepts per TR). Of the SVs, 14% (5/35) contained 94.59% (6157/6509) of all the concepts. The SVs were International Classification of Diseases, Tenth Revision Procedure Coding System (5479/6509, 84.18% of concepts), Systematized Nomenclature of Medicine-Clinical Terms (United States; 326/6509, 5.01% of concepts), Current Procedural Terminology (142/6509, 2.18% of concepts), MedDRA (115/6509, 1.77% of concepts), and MEDCIN (95/6509, 1.46% of concepts). The remaining 5.41% (352/6509) of concepts were contained in 86% (30/35) of SVs. All data are presented in Multimedia Appendix 1.

The SVs were created by 29 individual authors. The US National Library of Medicine was the author of (4/29, 14%) SVs. The National Center for Health Statistics and the Centers for Medicare and Medicaid Services, National Cancer Institute Enterprise Vocabulary Services, and the College of American Pathologists or International Health Terminology Standards Development Organization contributed 7% (2/35) of SVs each. Other 26 authors contributed with 3% (1/35) SV each. An overview of SVs is presented in Multimedia Appendix 2.

### Vocabulary Sources

The authors of the SVs were predominantly legal entities registered in the United States (25/35, 71%), followed by international organizations (6/35, 17%) and legal entities registered in Australia (2/35, 6%), the Netherlands, and the United Kingdom (1/35, 3%). Of the total SVs, (16/35, 46%) did not have any restriction on use, whereas the remaining (19/35, 54%) SVs had some level of restriction. Of the 35 SVs, 20 (57%) were updated within the last 5 years (Multimedia Appendix 3).

All concepts found in RT SVs were labeled with one of 29 semantic types (Multimedia Appendix 4), which accounted for 21.8% (29/133) of all semantic types available in UMLS. Of the total concepts, 94.05% (6122/6509) were classified by UMLS as terms describing a *therapeutic or preventive procedure* according to the scheme for classification of semantic types.

After removing the *stop words*, the total number of words for all SVs was 56,219, with a median of 25 unique words per TR (range 3-50,682). The total number of unique words in all SVs was 1048, with a median of 19 unique words per vocabulary (range 3-406). The lexical density for all concepts in all SVs was zero (0.02 rounded to 2 decimals; Multimedia Appendix 5). Median lexical density per unique TR was 0.7 (range 0.0-1.0). The median maximal length of all concepts expressed as the total number of words for all TR was 8 (range, 3-28). The median minimal length of all concepts expressed as the total number of words for all TRs was 3 (range 1-10). All results are shown in Table 1.

Of the total words, 677 were unique to only one TR, whereas four words were present in multiple SVs: *radiation* was present in 31, *therapy* in 28, *radiotherapy* in 18, and *procedure* in 11 SVs. We did not identify any TR specifically dedicated to radiation therapy.

**Table 1:** Properties of source vocabularies.

Vocabulary resource	Total number of all words	Total number of unique words	Lexical density of the vocabulary resource	Average length of the concept	Median length of the concept	Maximal length of the concept	Minimal length of the concept
ATC^a^	5	5	1.0	5	5	5	5
CCS^b^	3	3	1.0	3	3	3	3
CHV^c^	3	3	1.0	3	3	3	3
ICD10AMAE^d^	12	12	1.0	6	6	7	5
ICD9CM^e^	7	7	1.0	4	4	5	2
MTHICD9^f^	9	9	1.0	9	9	9	9
NANDA-I^g^	4	4	1.0	4	4	4	4
NCI_CPTAC^h^	3	3	1.0	3	3	3	3
SNM^i^	7	7	1.0	7	7	7	7
PCDS^j^	9	8	0.9	9	9	9	9
NIC^k^	34	30	0.9	11	8	18	8
ICD10CM^l^	9	7	0.8	5	5	5	4
ALT^m^	25	19	0.8	8	8	14	3
ICD-10^n^	25	19	0.8	6	5	14	2
CCPSS^o^	7	5	0.7	4	4	4	3
ICNP^p^	6	4	0.7	3	3	3	3
PDQ^q^	27	18	0.7	5	6	8	3
ICD10AM^r^	29	19	0.7	6	6	7	4
ICPC2ICD10ENG^s^	19	12	0.6	3	2	5	2
MSH^t^	52	32	0.6	3	3	4	2
SPN^u^	24	14	0.6	5	5	6	4
CSP^v^	7	4	0.6	4	4	4	3
HCPCS^w^	97	54	0.6	14	13	22	10
SNMI^x^	71	36	0.5	6	7	8	5
MTH^y^	265	132	0.5	4	4	23	1
HL7V3.0^z^	46	20	0.4	7	6	10	4
NCI Thesaurus	279	115	0.4	4	4	14	1
Read Codes	61	22	0.4	4	3	5	3
MEDCIN	488	175	0.4	5	5	10	2
UMD^aa^	312	97	0.3	5	5	9	2
SNOMEDCT_US^ab^	1420	406	0.3	4	4	10	2
LOINC^ac^	365	96	0.3	8	8	19	2
MedDRA^ad^	409	84	0.2	4	4	8	2
Current Procedural Terminology	1398	228	0.2	10	9	28	3
ICD10PCS^ae^	50,682	175	0.0	9	9	15	2
Median	25	19	0.7	5	5	8	3
Minimal	3	3	0.0	3	2	3	1
Maximal	50,682	406	1.0	14	13	28	10

^a^ATC: Anatomical Therapeutic Chemical Classification System.

^b^CCS: Clinical Classifications Software.

^c^CHV: Consumer Health Vocabulary.

^d^ICD10AMAE: International Classification of Diseases and Related Health Problems, Tenth Revision, Australian Modification, Americanized English Equivalents.

^e^ICD9CM: International Classification of Diseases, Ninth Revision, Clinical Modification Entry Terms.

^f^MTHICD9: Metathesaurus Names International Classification of Diseases, Ninth Revision, Clinical Modification Entry Terms.

^g^NANDA-I: NANDA-I Taxonomy.

^h^NCI_CPTAC: Clinical Proteomic Tumor Analysis Consortium.

^i^SNM: The Systematized Nomenclature of Medicine, Second Edition.

^j^PCDS: Patient Care Data Set.

^k^NIC: Nursing Interventions Classification.

^l^ICD10CM: International Classification of Diseases, Tenth Revision, Clinical Modification.

^m^ALT: Alternative Billing Concepts.

^n^ICD-10: International Classification of Diseases, Tenth Revision.

^o^CCPSS: Clinical Problem Statements.

^p^ICNP: International Classification for Nursing Practice.

^q^PDQ: Physician Data Query.

^r^ICD10AM: International Classification of Diseases, Tenth Revision, Australian Modification.

^s^ICPC2ICD10ENG: International Classification of Primary Care-International Classification of Diseases, Tenth Revision Thesaurus.

^t^MSH: Medical Subject Headings.

^u^SPN: Standard Product Nomenclature.

^v^CSP: CRISP Thesaurus.

^w^HCPCS: Healthcare Common Procedure Coding System.

^x^SNMI: The Systemized Nomenclature of Human and Veterinary Medicine.

^y^MTH: Metathesaurus Names.

^z^HL7V3.0: Health Level Seven version 3.0.

^aa^UMD: Universal Medical Device Nomenclature System.

^ab^SNOMEDCT_US: The Systematized Nomenclature of Medicine-Clinical Terms, US Edition.

^ac^ LOINC: Logical Observations Identifiers, Names, Codes.

^ad^MedDRA: Medical Dictionary for Regulatory Activities.

^ae^ICD10PCS: The International Classification of Diseases, Tenth Revision, Procedure Coding System.

## Discussion

### Principal Findings

To the best of our knowledge, this is the first study to evaluate the state of standardized SVs dedicated to radiation therapy. Although our search of UMLS retrieved a large number of unique concepts distributed across 35 SVs, none of them was identified as a dedicated TR for radiation oncology. However, such a dedicated TR, providing standardized terms for modern radiation therapy and being widely adopted, is a prerequisite for achieving interoperability. The existing SVs are concentrated on describing different radiation therapy techniques, most probably for reimbursement coding purposes.

An important milestone in the standardization of radiation therapy communication was published by the International Commission on Radiation Units and Measurements report 50, 62, and 83. The International Commission on Radiation Units and Measurements 83 provides the information necessary to standardize techniques and procedures and harmonize the prescribing, recording, and reporting of intensity-modulated radiation therapy. The most significant achievements were made in the recommendation of the definition, selection, and delineation of the radiation therapy volumes along with dose prescription to the volumes and dose-volume reporting recommendations [22]. However, the International Commission on Radiation Units and Measurements 83 does not go beyond a high-level abstract and conceptual description of the target and risk volumes, whereas the specific anatomy of the patient was not subject to standardization.

Furthermore, the American Society for Radiation Oncology (ASTRO) and the American Association of Physicists in Medicine have recognized the need for a unified and standardized terminological approach to radiation therapy. Members of both societies published several papers and recommendations concerning standardized approaches in naming conventions for radiation therapy [13,20,26-28].

The authors of the ASTRO white paper, published in 2016, have argued that the standardized terminology approach in dose prescription will facilitate accurate communication among providers to support safe practice and guide product developers in creating software consistent with the best standard of practice [21]. To avoid common pitfalls of standardization efforts [29], the working group limited their efforts to standardize the central prescription items concerning how the prescribed dose is specified. They suggested standardization of key elements for prescription, such as treatment site, delivery method, dose per fraction, fraction number, total dose, and a special field named *other elements*. They commented on the previous work of other groups such as the American College of Radiology–ASTRO Practice Parameter for Radiation Oncology [28], the ASTRO Accreditation Program for Excellence Standard [27], and ASTRO recommendation for documenting intensity-modulated radiation therapy [26]. The authors of the white paper rightfully argued that although thoughtfully developed, the proposals will be hard to implement in the modern environment. Some items are not sufficiently precise, and other concepts are difficult to define as our field evolves.

The most challenging and intellectually demanding process is the formalization of treatment sites. Frequently, radiation therapy volumes span several distinct anatomical entities and consist of numerous anatomical regions. For example, treatment volumes for head and neck cancer traditionally consist of macroscopic tumors or former tumor sites that span several anatomical entities of the digestive tract and respiratory organs. In malignancies of pelvic origin, it is common to have part volumes extending to the abdominal region or lower extremities (eg, paraaortic volumes or partially in the upper extremities in vulvar cancer). Large tumors of any histology, such as sarcomas or metastatic diseases, sometimes create geometric forms that are very difficult to intuitively define using standard anatomical descriptors, such as lymph node levels or anatomical organ boundaries. An additional level of complexity is added through time-dependent changes in volume shapes and the introduction of subvolumes, which receive a different dose synchronously (eg, simultaneous integrated boost).

Further important work in the domain of RT vocabulary standardization has been done by the American Association of Physicists in Medicine Task Group 263. Their 2018 published report provides a detailed overview of scientific literature, previous achievements, contemporary practice, and some future directions related to nomenclature standardization [20]. The main output of this report is the development of a nomenclature system for target volumes, organs at risk, and dose-volume histogram metrics with the goal of straightforward adoption in current practice. In contrast to all previous initiatives for structure standardization, this nomenclature was developed by an assembly of stakeholders in radiation oncology, including multiple societies (eg, ASTRO, the European Society for Radiotherapy and Oncology, and others), disciplines, and vendors, ensuring broad endorsement and use of the nomenclature. A major drawback of this approach was the intention to primarily accommodate and serve the manufacturers of radiation therapy software and hardware, and not patients or physicians. By doing so, we are risking repeating the major historical mistakes, which have brought us to our current position. Patients, physicians, or payers must have clearer and understandable naming conventions designed according to their needs.

In this light, we must consider the newest development in the legislative environment in the United States formalized in the 21st-Century Cures Act, signed into law on December 13, 2016, which is designed to help accelerate medical product development and bring innovations and advances to patients who need them faster and more efficiently. The act finally results in the ONC’s Cures Act Final Rule, which supports seamless and secure access, exchange, and use of eHealth information. However, good initiatives and their formulation within legal boundaries sometimes collide with reality. The last surge of digitalization in the United States ended in the complete lockdown of information within vendor software. Furthermore, this led to a phenomenon popularly known as a *death by a thousand clicks* [5], which resulted in a health care crisis and unprecedented burnout rate among physicians [8].

We acknowledge the limitations of this study. As the most important limitation, we acknowledge the missing review of ontological repositories such as BioPortal or OBO Foundry. Furthermore, we reviewed only UMLS. It is possible that there are some TRs in other languages that we are not aware of. Despite this limitation, we believe that this analysis provides a realistic overview of the current state of terminologies developed specifically for radiation oncology. Cross-sectional analysis is important, even if negative.

### Conclusions

Cancer is still one of the leading causes of death and morbidity globally, and oncological research comprises approximately one-quarter of the complete biomedical clinical research portfolio [30]. Radiation oncology will be used in at least 50% of cancer patients for treatment or palliation, is an important contributor to survival and symptom control, and is an essential part of streaming toward precise and personalized medicine [31,32]. However, without meaningful digitalization and high data availability, we may not achieve the desired effects. To achieve the promise of digitalization in the clinical environment, we need SI in practice [1]. The basis for SI is shared TR. We need to establish an agile, productive, and progressive way for communication among all actors in radiation therapy and beyond through the development of dedicated radiation therapy-specific virtual reality.

## Appendix

Multimedia Appendix 1 A complete database used for analysis.

Multimedia Appendix 2 Overview of the source vocabularies.

Multimedia Appendix 3 A number of lexical resources published according to last year’s update.

Multimedia Appendix 4 A number of terms per Unified Medical Language System semantic type category.

Multimedia Appendix 5 A number of words per source vocabulary.

## Abbreviations

ASTRO: American Society for Radiation Oncology
SI: semantic interoperability
SV: source vocabulary
TR: terminology resource
UMLS: Unified Medical Language System
