# Supplementary material for: Toward Data-Driven Radiation Oncology Using Standardized Terminology as a Starting Point: Cross-sectional Study
Source: JMIR Form Res. 2022 Jan 19;6(1):e27550. doi: 10.2196/27550 (PMC8811690; doi:10.2196/27550)
Supplement: Multimedia Appendix 2 [file formative_v6i1e27550_app2.docx]

**Table 1.** Overview of the source vocabularies.

| UMLS^a^ abbreviation | Name of the vocabulary resource | Name of the publisher | Country of origin | Last update | Licensing restriction level as defined in UMLS |
| --- | --- | --- | --- | --- | --- |
| ALT | ABC^b^ | ABC coding solutions | United States | 2009 | 3 |
| ATC | Anatomical Therapeutic Chemical Classification System | World Health Organization Collaborating Center for Drug Statistics Methodology | International Organization | 2020 | 0 |
| CCPSS | Clinical Problem Statements | Vanderbilt University or Emory University Department of Medicine and Medical Systems Development Corporation | United States | 2000 | 3 |
| CCS | Clinical Classification Software | Healthcare Cost and Utilization Project | United States | 2005 | 0 |
| CHV | Consumer Health Vocabulary | Biomedical Informatics Department at the University of Utah | United States | 2012 | 0 |
| CPT | Current Procedural Terminology | American Medical Association | United States | 2020 | 3 |
| CSP | CRISP Thesaurus | National Institutes of Health | United States | 2006 | 0 |
| HCPCS | Healthcare Common Procedure Coding System | CMS^c^ | United States | 2020 | 0 |
| HL7V3.0 | HL7 version 3.0 | Health Level Seven international | International organization | 2020 | 0 |
| ICD-10 | ICD10^d^ | World Health Organization | International organization | 2004 | 3 |
| ICD10AM | ICD-10, Australian Modification | Australian NCCH^e^ | Australia | 2000 | 3 |
| ICD10AMAE | ICD-10, Australian Modification, Americanized English Equivalents | NCCH University of Sydney Faculty of Health Sciences | Australia | 2002 | 3 |
| ICD10CM | ICD-10, Clinical Modification | NCHS^f^ and the CMS | United States | 2019 | 4 |
| ICD10PCS | ICD-10 Procedure Coding System | CMS | United States | 2019 | 0 |
| ICD9CM | International Classification of Diseases, Ninth Revision, Clinical Modification | NCHS and the CMS | United States | 2015 | 0 |
| ICNP | ICN^g^ Practice | ICN | International organization | 2019 | 3 |
| ICPC2ICD10ENG | ICPC2-ICD10 Thesaurus | Transition Project Foundation Amsterdam | Netherland | 2005 | 3 |
| LNC | LOINC | Regenstrief Institute, Inc (Indiana University) | United States | 2020 | 0 |
| MDR | MedDRA | International Conference on Harmonization | International organization | 2020 | 3 |
| MEDCIN | MEDCIN | Medicomp Systems, Inc | United States | 2020 | 3 |
| MSH | MeSH^h^ | US NLM^i^ | United States | 2020 | 0 |
| MTH | Metathesaurus Names | US NLM | United States | 1990 | 0 |
| MTHICD9 | Metathesaurus Names ICD-9-CM^j^ Entry Terms | US NLM | United States | 2015 | 0 |
| NANDA-I | NANDA-I Taxonomy | NANDA International, Inc | International organization | 2018 | 3 |
| NCI | NCI^k^ Thesaurus | NCI EVS^l^ | United States | 2020 | 0 |
| NCI_CPTAC | Clinical Proteomic Tumor Analysis Consortium | NCI EVS | United States | 2019 | 0 |
| NIC | Nursing Interventions Classification | University of Iowa College of Nursing | United States | 2018 | 3 |
| PCDS | Patient Care Data Set | Judy Ozbolt at the University of Virginia, University Health System Consortium | United States | 1999 | 3 |
| PDQ | Physician Data Query | US NCI | United States | 2019 | 0 |
| RCD | Read Codes | The United Kingdom. National Health Service Center for Coding and Classification | United Kingdom | 2000 | 3 |
| SNM | SNOMED^m^ 1982 | College of American Pathologists or International Health Terminology Standards Development Organization | United States | 1991 | 9 |
| SNMI | SNOMED Intl 1998 | College of American Pathologists or International Health Terminology Standards Development Organization | United States | 1999 | 9 |
| SNOMEDCT_US | SNOMED-Clinical Terms, United States Edition | US NLM | United States | 2020 | 9 |
| SPN | Standard Product Nomenclature | CDRH at the United States FDA^n^ | United States | 2004 | 0 |
| UMD | UMDNS | ECRI institute | United States | 2020 | 1 |

^a^UMLS: Unified Medical Language System.

^b^ABC: Alternative Billing Concepts.

^c^CMS: Centers for Medicare and Medicaid Services.

^d^ICD-10: International Classification of Diseases, Tenth Revision.

^e^NCCH: National Center for Classification in Health.

^f^NCHS: National Center for Health Statistics.

^g^ICN: International Classification for Nursing.

^h^MeSH: Medical Subject Headings.

^i^NLM: National Library of Medicine.

^j^ICD-9-CM: International Classification of Diseases, Ninth Revision, Clinical Modification.

^k^NCI: National Cancer Institute.

^l^EVS: Enterprise Vocabulary Services.

^m^SNOMED: Systematized Nomenclature of Medicine.

^n^FDA: Food and Drugs Administration.

**.** Overview of the source vocabularies.

| UMLS^a^ abbreviation | Name of the vocabulary resource | Name of the publisher | Country of origin | Last update | Licensing restriction level as defined in UMLS |
| --- | --- | --- | --- | --- | --- |
| ALT | ABC^b^ | ABC coding solutions | United States | 2009 | 3 |
| ATC | Anatomical Therapeutic Chemical Classification System | World Health Organization Collaborating Center for Drug Statistics Methodology | International Organization | 2020 | 0 |
| CCPSS | Clinical Problem Statements | Vanderbilt University or Emory University Department of Medicine and Medical Systems Development Corporation | United States | 2000 | 3 |
| CCS | Clinical Classification Software | Healthcare Cost and Utilization Project | United States | 2005 | 0 |
| CHV | Consumer Health Vocabulary | Biomedical Informatics Department at the University of Utah | United States | 2012 | 0 |
| CPT | Current Procedural Terminology | American Medical Association | United States | 2020 | 3 |
| CSP | CRISP Thesaurus | National Institutes of Health | United States | 2006 | 0 |
| HCPCS | Healthcare Common Procedure Coding System | CMS^c^ | United States | 2020 | 0 |
| HL7V3.0 | HL7 version 3.0 | Health Level Seven international | International organization | 2020 | 0 |
| ICD-10 | ICD10^d^ | World Health Organization | International organization | 2004 | 3 |
| ICD10AM | ICD-10, Australian Modification | Australian NCCH^e^ | Australia | 2000 | 3 |
| ICD10AMAE | ICD-10, Australian Modification, Americanized English Equivalents | NCCH University of Sydney Faculty of Health Sciences | Australia | 2002 | 3 |
| ICD10CM | ICD-10, Clinical Modification | NCHS^f^ and the CMS | United States | 2019 | 4 |
| ICD10PCS | ICD-10 Procedure Coding System | CMS | United States | 2019 | 0 |
| ICD9CM | International Classification of Diseases, Ninth Revision, Clinical Modification | NCHS and the CMS | United States | 2015 | 0 |
| ICNP | ICN^g^ Practice | ICN | International organization | 2019 | 3 |
| ICPC2ICD10ENG | ICPC2-ICD10 Thesaurus | Transition Project Foundation Amsterdam | Netherland | 2005 | 3 |
| LNC | LOINC | Regenstrief Institute, Inc (Indiana University) | United States | 2020 | 0 |
| MDR | MedDRA | International Conference on Harmonization | International organization | 2020 | 3 |
| MEDCIN | MEDCIN | Medicomp Systems, Inc | United States | 2020 | 3 |
| MSH | MeSH^h^ | US NLM^i^ | United States | 2020 | 0 |
| MTH | Metathesaurus Names | US NLM | United States | 1990 | 0 |
| MTHICD9 | Metathesaurus Names ICD-9-CM^j^ Entry Terms | US NLM | United States | 2015 | 0 |
| NANDA-I | NANDA-I Taxonomy | NANDA International, Inc | International organization | 2018 | 3 |
| NCI | NCI^k^ Thesaurus | NCI EVS^l^ | United States | 2020 | 0 |
| NCI_CPTAC | Clinical Proteomic Tumor Analysis Consortium | NCI EVS | United States | 2019 | 0 |
| NIC | Nursing Interventions Classification | University of Iowa College of Nursing | United States | 2018 | 3 |
| PCDS | Patient Care Data Set | Judy Ozbolt at the University of Virginia, University Health System Consortium | United States | 1999 | 3 |
| PDQ | Physician Data Query | US NCI | United States | 2019 | 0 |
| RCD | Read Codes | The United Kingdom. National Health Service Center for Coding and Classification | United Kingdom | 2000 | 3 |
| SNM | SNOMED^m^ 1982 | College of American Pathologists or International Health Terminology Standards Development Organization | United States | 1991 | 9 |
| SNMI | SNOMED Intl 1998 | College of American Pathologists or International Health Terminology Standards Development Organization | United States | 1999 | 9 |
| SNOMEDCT_US | SNOMED-Clinical Terms, United States Edition | US NLM | United States | 2020 | 9 |
| SPN | Standard Product Nomenclature | CDRH at the United States FDA^n^ | United States | 2004 | 0 |
| UMD | UMDNS | ECRI institute | United States | 2020 | 1 |

^a^UMLS: Unified Medical Language System.

^b^ABC: Alternative Billing Concepts.

^c^CMS: Centers for Medicare and Medicaid Services.

^d^ICD-10: International Classification of Diseases, Tenth Revision.

^e^NCCH: National Center for Classification in Health.

^f^NCHS: National Center for Health Statistics.

^g^ICN: International Classification for Nursing.

^h^MeSH: Medical Subject Headings.

^i^NLM: National Library of Medicine.

^j^ICD-9-CM: International Classification of Diseases, Ninth Revision, Clinical Modification.

^k^NCI: National Cancer Institute.

^l^EVS: Enterprise Vocabulary Services.

^m^SNOMED: Systematized Nomenclature of Medicine.

^n^FDA: Food and Drugs Administration.
