# Supplementary figures and images for: Toward Data-Driven Radiation Oncology Using Standardized Terminology as a Starting Point: Cross-sectional Study
Source: JMIR Form Res. 2022 Jan 19;6(1):e27550. doi: 10.2196/27550 (PMC8811690; doi:10.2196/27550)

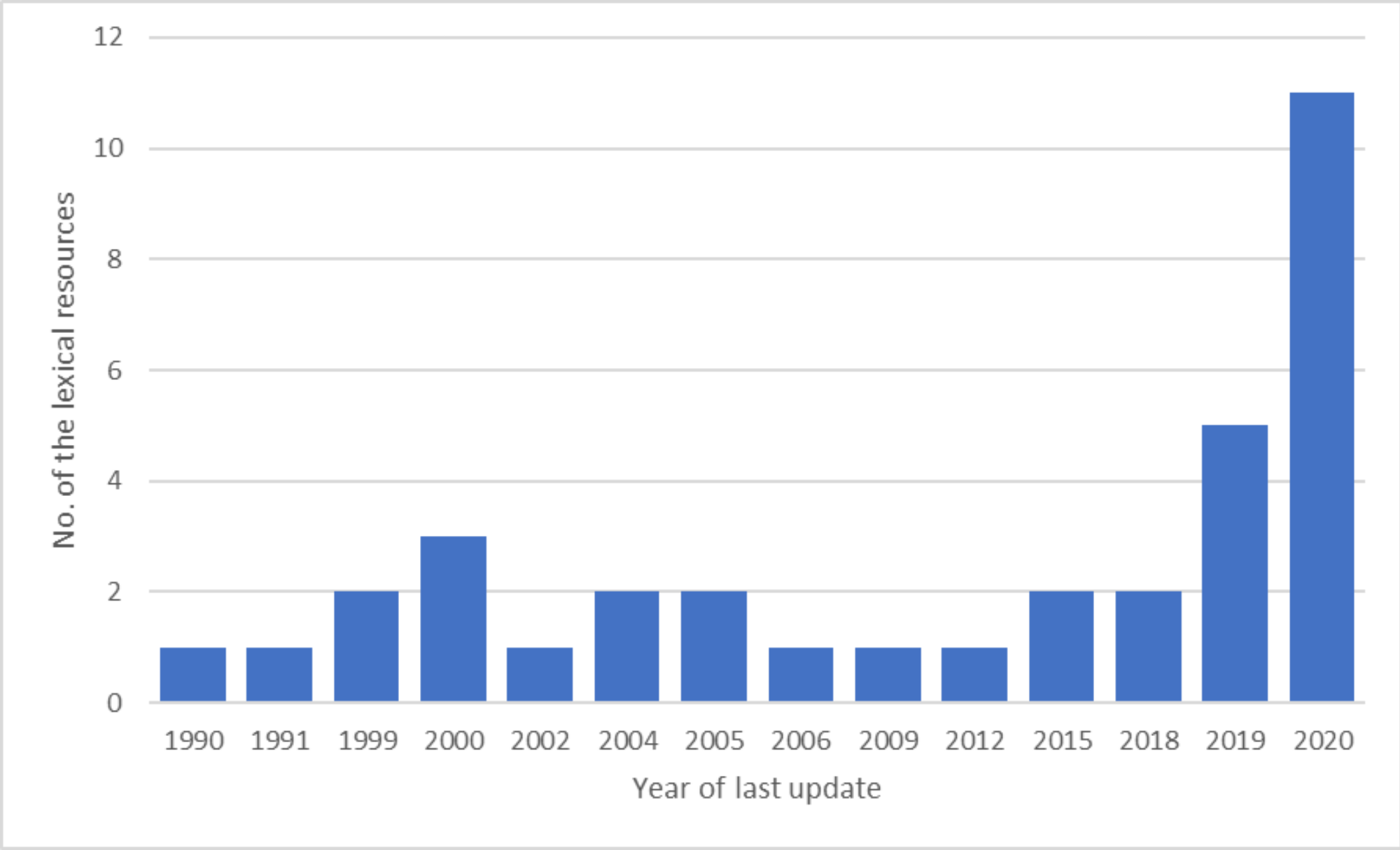

Supplement: Multimedia Appendix 3 [file formative_v6i1e27550_app3.docx]

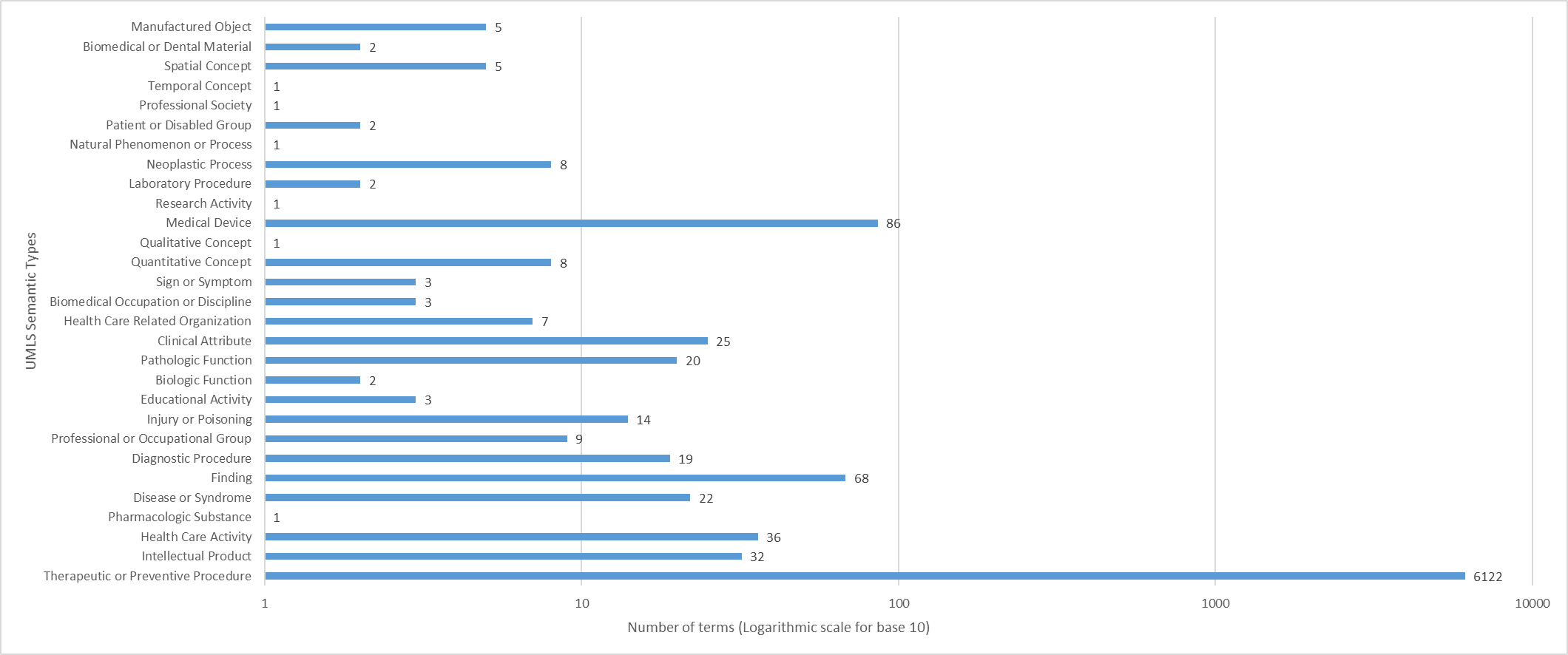

Supplement: Multimedia Appendix 4 [file formative_v6i1e27550_app4.docx]

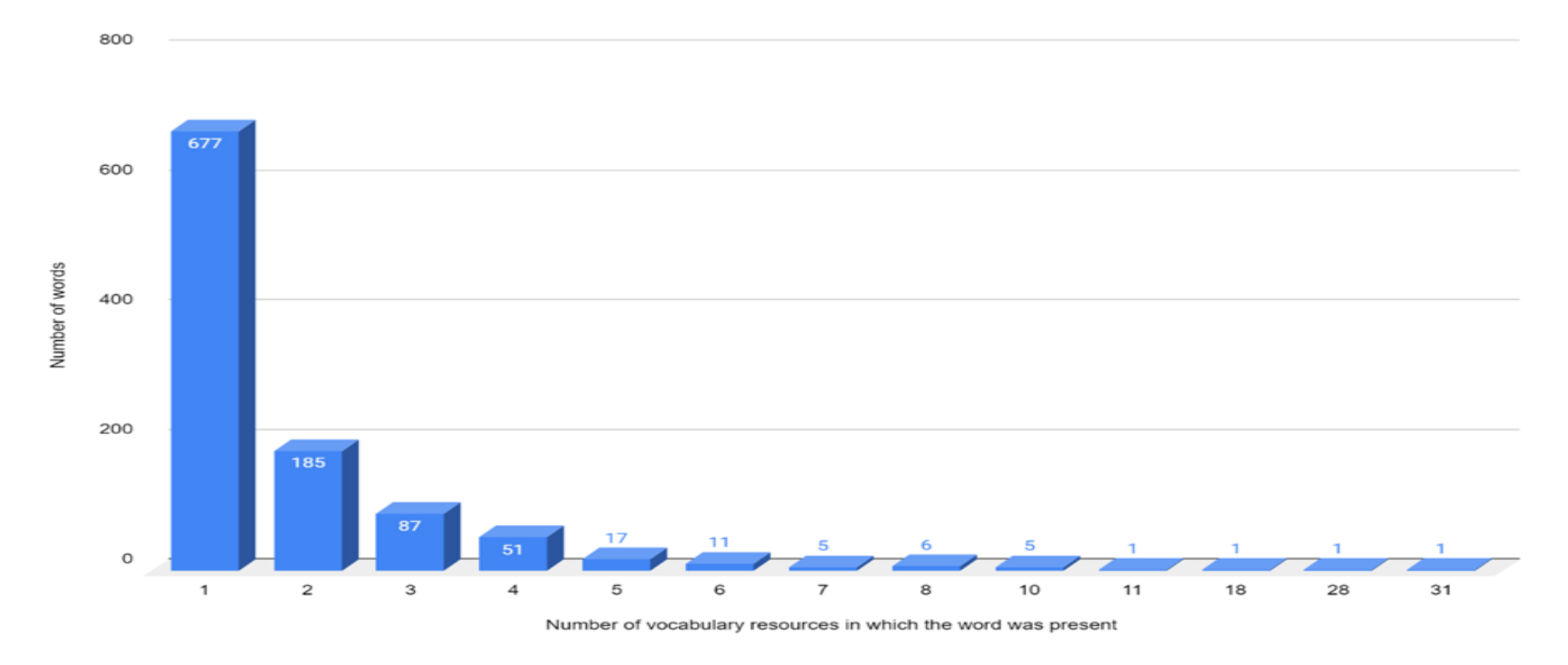

Supplement: Multimedia Appendix 5 [file formative_v6i1e27550_app5.docx]
